# Supplementary material for: Mapping the structure-function relationship along macroscale gradients in the human brain
Source: Nat Commun. 2024 Aug 16;15:7063. doi: 10.1038/s41467-024-51395-6 (PMC11329792; doi:10.1038/s41467-024-51395-6)
Supplement: Supplementary file 1 — Supplementary Information [file 41467_2024_51395_MOESM1_ESM.pdf]

Supplementary Information

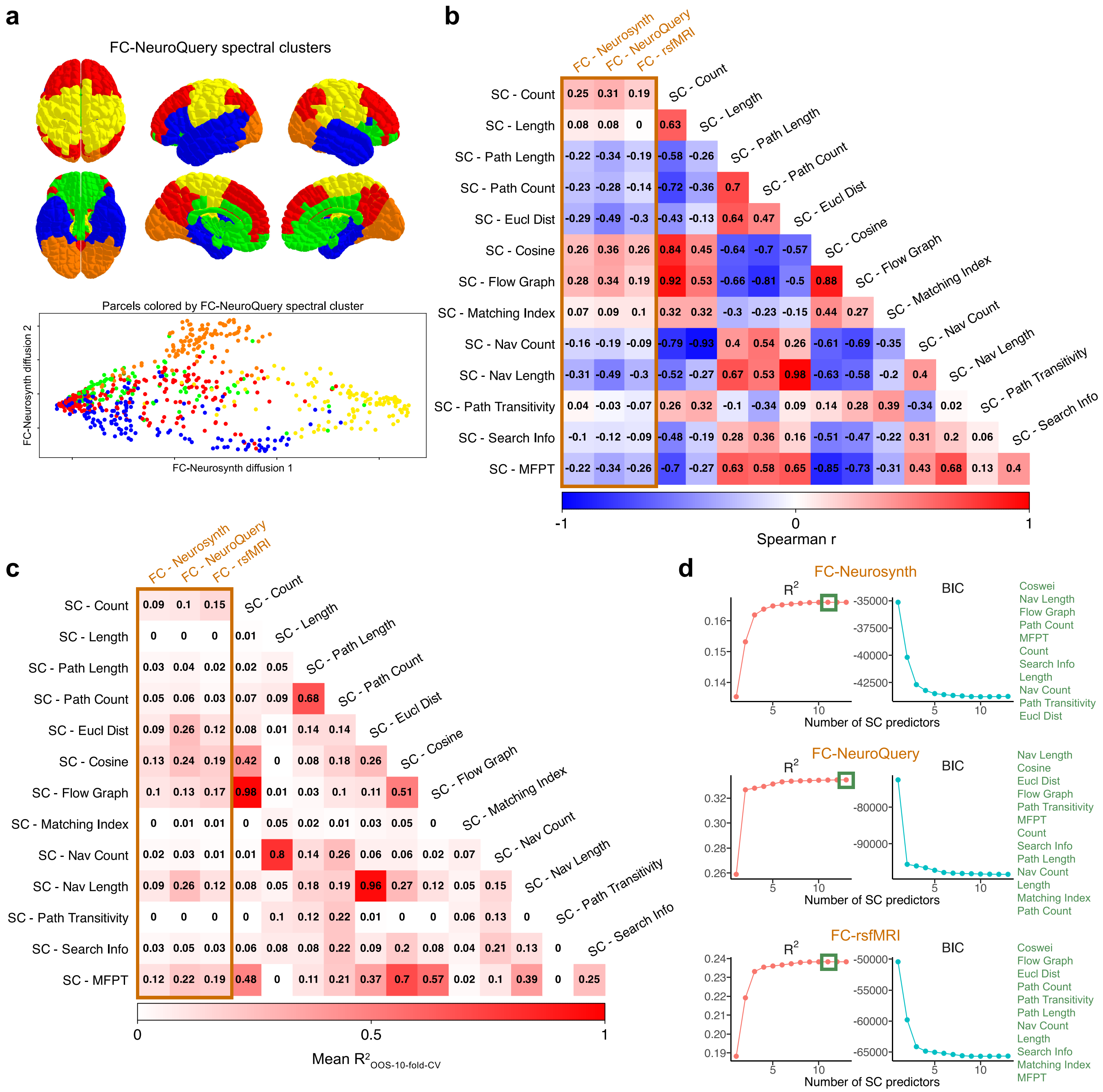

Fig. S1: Additional results for global comparison between structural and functional connectivities. **a** Spectral clustering of parcels based on FC-NeuroQuery (top). Two-dimensional diffusion map for FC-Neurosynth with each parcel colored according to FC-NeuroQuery spectral cluster designation (bottom). Analogous to Fig. 3b-c. **b** Spearman *r* values from correlation tests between specific SC and FC values across the whole brain. All estimates within the gold box represent structure-function relationships. **c** Mean out-of-sample adjusted R^2 values across 10 cross-validation folds from linear regression models evaluating the relationship between pairs of SC or FC values across the whole brain. The cross-validation folds are identical to those used in Fig. 2e. All estimates within the gold box represent structure-function relationships. Minor differences are exhibited between this figure and Fig. 2d, which shows in-sample R^2

values across all data. **d** Adjusted in-sample R^2 values from linear regression models to predict either FC-Neurosynth, FC-NeuroQuery, or FC-rsfMRI with varying number of SC predictors. Comparable plots for Bayesian information criterion (BIC) are also shown. For each number of SC predictors, the optimal model was selected via exhaustive search. The list of terms in green reflect the optimal SC predictors that maximize adjusted R^2, beyond which adding more SC predictors does not improve performance for that FC type. Points denote mean values and error bars denote +/- SEM. Because this approach can lead to overfitting, the models illustrated in Fig. 2e were ultimately used instead. Source data are provided as a Source Data file.

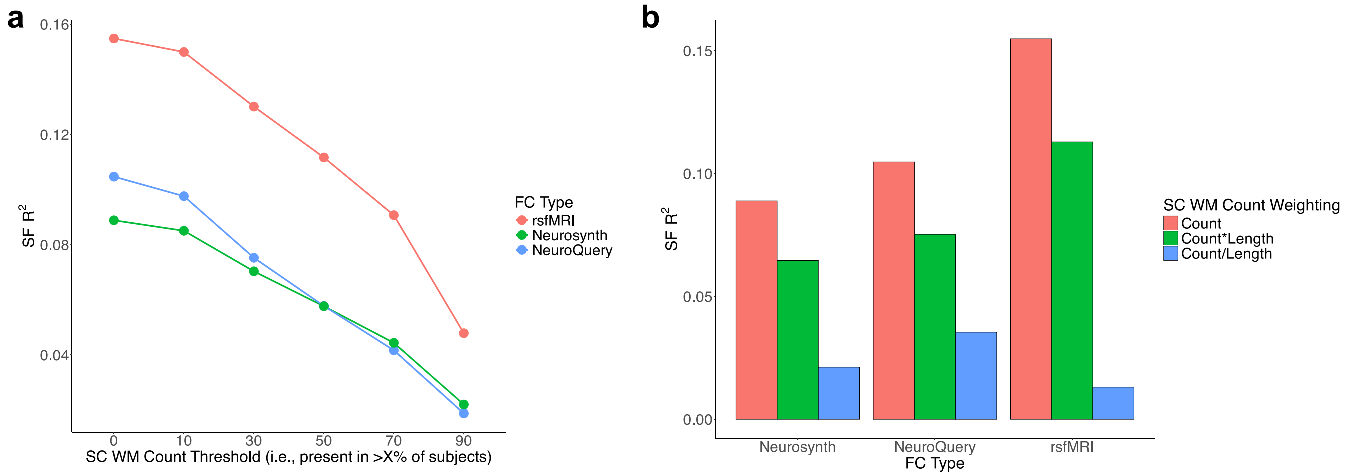

Fig. S2: Structure-function correspondence is maximized without any zeroing threshold or weighting for SC-Count. a Different SC-Count zeroing thresholds (i.e., any connection must have nonzero WM streamlines in more than some X% of subjects for inclusion in the group-level matrix) were evaluated for their R^2 values resulting from linear regression models of FC-rsfMRI, FC-Neurosynth, and FC-NeuroQuery. The highest structure-function correspondence can be seen to occur when no zeroing threshold is applied to the SC-Count data. **b** Different SC-Count weights (i.e., either multiplying or dividing by WM length) were evaluated for their R^2 values resulting from linear regression models of FC-rsfMRI, FC-Neurosynth, and FC-NeuroQuery. The highest structure-function correspondence can be seen to occur when no weighting is applied to the SC-Count data. Source data are provided as a Source Data file.

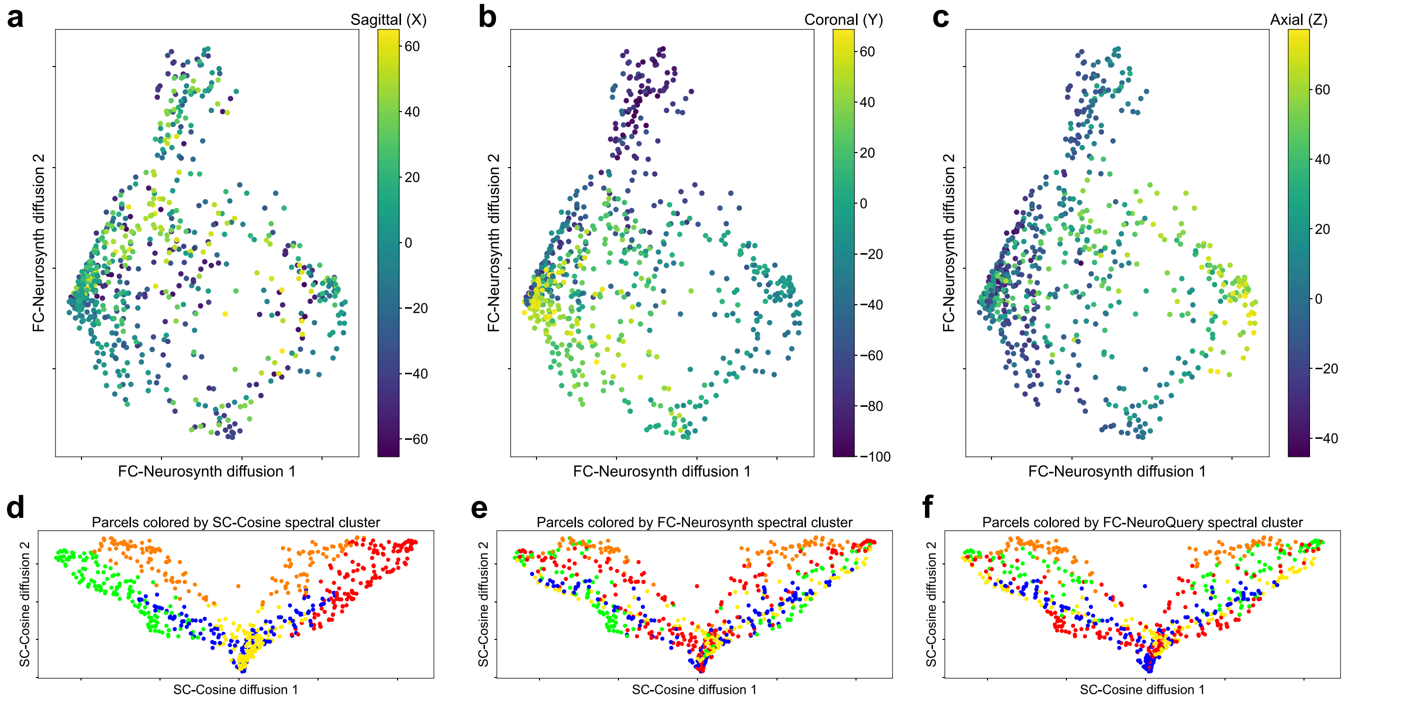

Fig. S3: Cartesian coordinates separate parcels in functional diffusion map, and visualization of structural diffusion map aids comparison of SC and FC spectral clusterings. Two-dimensional diffusion map for FC-Neurosynth with each parcel colored according to **a** sagittal, **b** coronal, and **c** axial Cartesian coordinates. When plotted on the two-dimensional FC diffusion map, the coronal and axial coordinates separate parcels well, suggesting a correlated continuum of function along these Cartesian dimensions in the brain. The sagittal coordinates did not separate parcels well in the FC diffusion map, likely due to lateralization. **d** Two-dimensional diffusion map for SC-Cosine with each parcel colored according to SC-Cosine spectral cluster designation. **e** Two-dimensional diffusion map for SC-Cosine with each parcel colored according to FC-Neurosynth spectral cluster designation. **f** Two-dimensional diffusion map for SC-Cosine with each parcel colored according to FC-NeuroQuery spectral cluster designation. Source data are provided as a Source Data file.

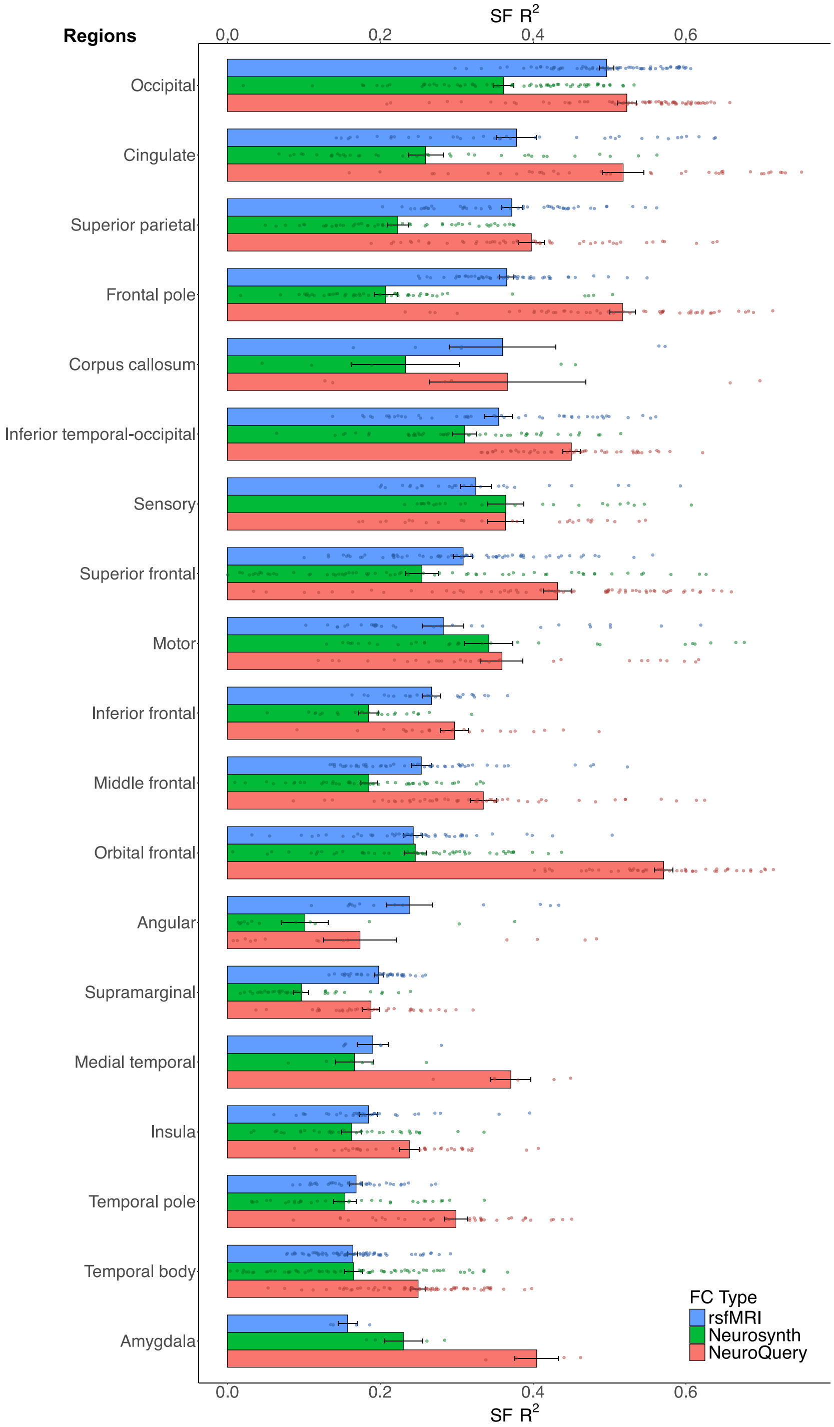

Fig. S4: Structure-function R^2 values to assess how structure-function correspondence varies by localization and differs depending on FC type. Analogous to Fig. 3D but using SF R^2 values instead of SF R^2 z-score values. Each bar represents the mean +/- SEM among constituent parcels for a particular region (N = 19). Each point (total N = 696) reflects a constituent parcel for that region. Source data are provided as a Source Data file.

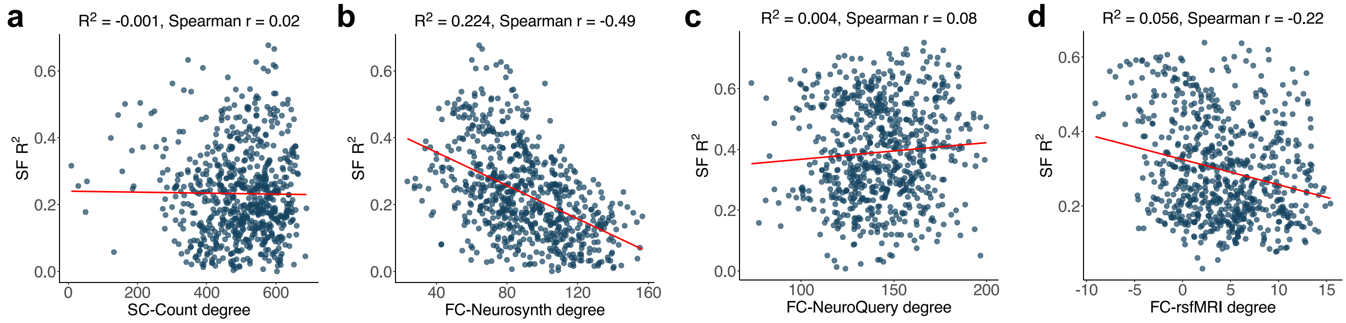

Fig. S5: Relationships between structure-function R^2 and structural and functional connectivity degrees. a Insignificant relationship between SF R^2 and SC-Count degree, suggesting the total number of emanating WM streamlines per parcel had minimal effect on SF correlation. **b** Significant relationship between SF R^2 and degree of FC-Neurosynth, suggesting parcels that are functionally similar in Neurosynth to many other parcels had lower SF correlation. **c** Insignificant relationship between SF R^2 and degree of FC-NeuroQuery, suggesting parcels that are functionally similar in NeuroQuery to many other parcels did not have significantly higher or lower SF correlation. **d** Marginally significant relationship between SF R^2 and degree of FC-rsfMRI, suggesting parcels that are functionally similar in resting state to many other parcels had slightly lower SF correlation. R^2 and Spearman *r* values are noted for all plots. Source data are provided as a Source Data file.

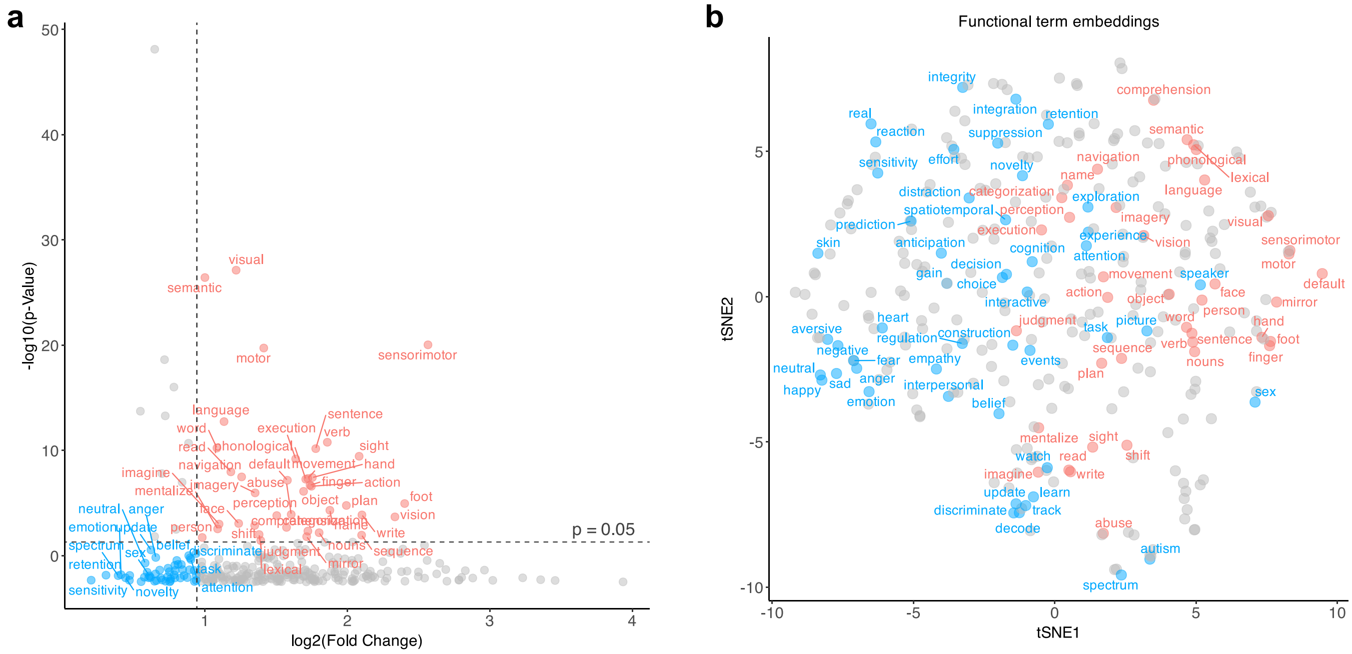

Fig. S6: Labelled functional terms with high and low structure-function correspondence. a Similar to Fig. 4b but with all high SF functional terms and select low SF functional terms labelled. **b** Similar to Fig. 4c but with all high SF and low SF functional terms labelled. Source data are provided as a Source Data file.

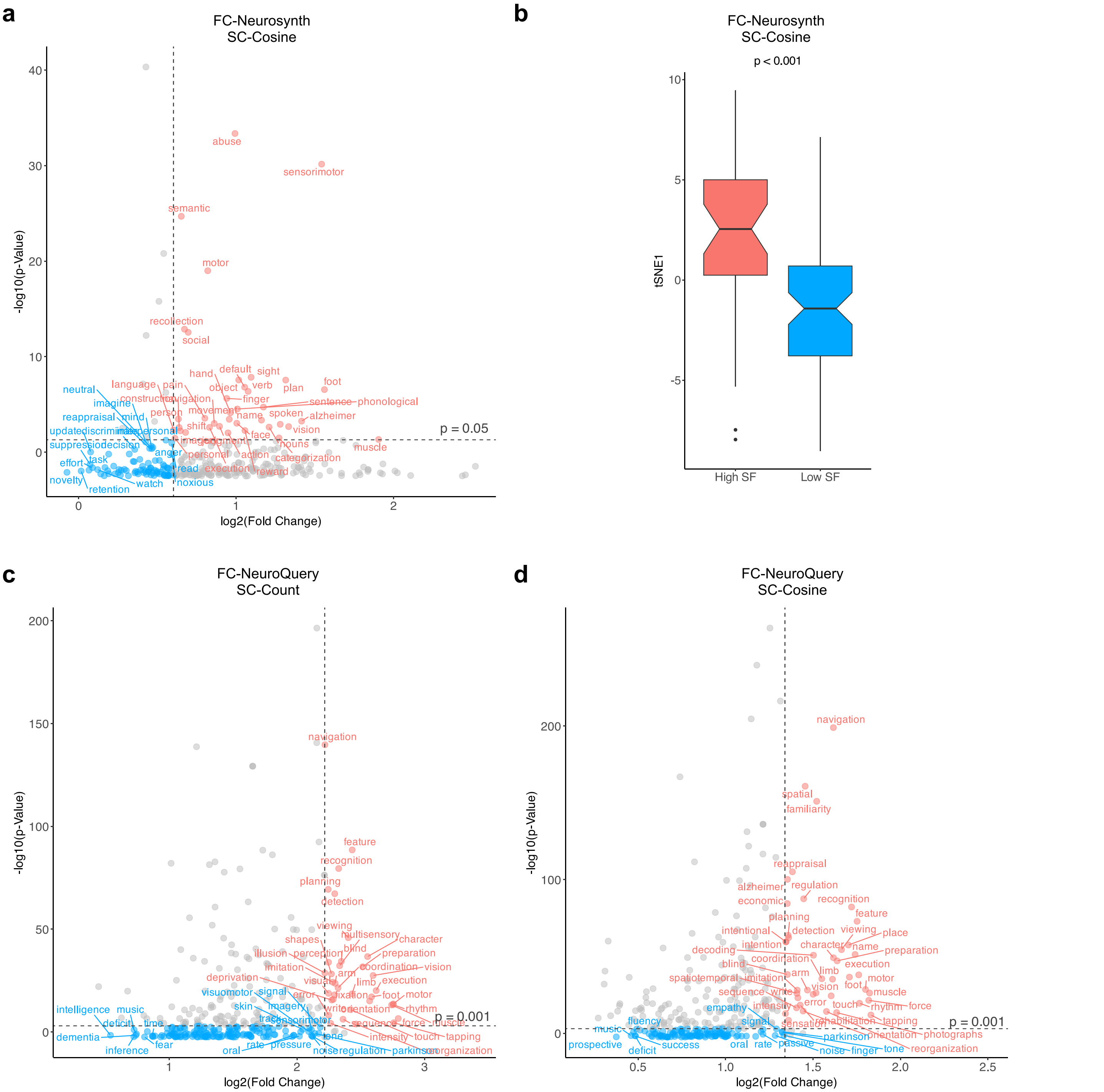

Fig. S7: Using alternative FC and SC types to discriminate between high and low structure-function correspondence functional terms reveals similar patterns. In addition to Fig. 4b and Fig. S5 which use FC-Neurosynth and SC-Count data, additional FC and SC types were used to map structure-function correspondence of functional terms. SC type selection directly affects both fold change and *p* value, whereas FC type selection indirectly does so by determining which parcels are active for each functional term. **a** Similar to Fig. S5a but with FC-Neurosynth and SC-Cosine used. **b** Similar to Fig. 4d but with FC-Neurosynth and SC-Cosine used, again demonstrating significant fundamental differences in embedding space between high SF (N = 37) and low SF (N = 80) functional terms. Two-sided Wilcoxon

signed-rank test was performed; exact p value is 1.39×10^{-6}. The boxplot has a box that signifies the interquartile range (IQR; 25th percentile to 75th percentile), a center bar that denotes the median, whiskers that extend up to 1.5 × IQR, and a notch that extends 1.58 × IQR / $\sqrt{n}$, where n is the sample size for that condition, to estimate the 95% confidence interval. **c** Similar to Fig. S5a but with FC-NeuroQuery and SC-Count used. Note for FC-NeuroQuery, we applied significance thresholds at Bonferroni-corrected p = 0.001 and $\log_2$(Fold Change) ≈ 1.79, reflecting the 80th percentile cutoff among significant terms. **d** Similar to Fig. S5a but with FC-NeuroQuery and SC-Cosine used. Note that additional NLP analyses were not conducted for these FC-NeuroQuery-derived SF correspondences to prevent issues with statistical dependence, as NeuroQuery uses word embeddings to smooth activation data. Source data are provided as a Source Data file.

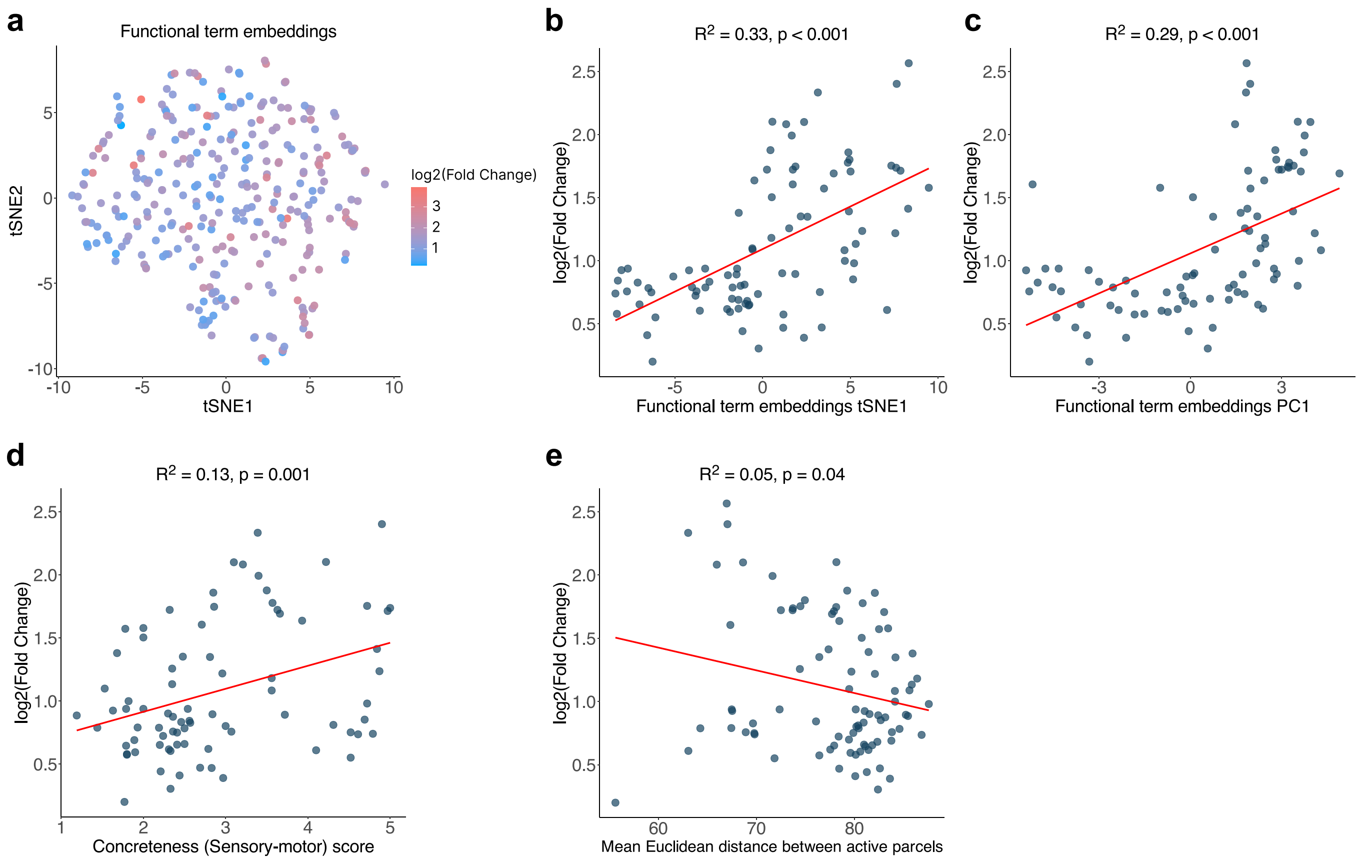

Fig. S8: Continuous representation of structure-function correspondence by term. Rather than categorizing functional terms as either high SF or low SF as done in Fig. 4, $\log_2$(Fold Change), which results from the Wilcoxon rank-signed test shown in Fig. 4a, was used as a continuous measure of SF correspondence by term. **a** Identical to Fig. 4c but colored according to $\log_2$(Fold Change). Analogous to Fig. 4d, there is a positive correlation between $\log_2$(Fold Change) and **b** the first tSNE component (exact p value of 5.46×10^{-9}), as well as **c** the first principal component of functional term word embeddings (exact p value of 5.77×10^{-8}). R^2 and p values are shown. **d** Analogous to Fig. 4e, there is a positive correlation between $\log_2$(Fold Change) and concreteness score, a proxy measure of the extent of sensory-motor function. R^2 and p values are shown. **e** To assess if SF correspondence simply scales with

proximity of parcels activated for a specific functional term, $\log_2$(Fold Change) values and the mean distance between active parcels are plotted for each term. The weak relationship reinforces that more complex factors than Euclidean distance better predict SF correspondence by functional term. R^2 and p values are shown. Source data are provided as a Source Data file.

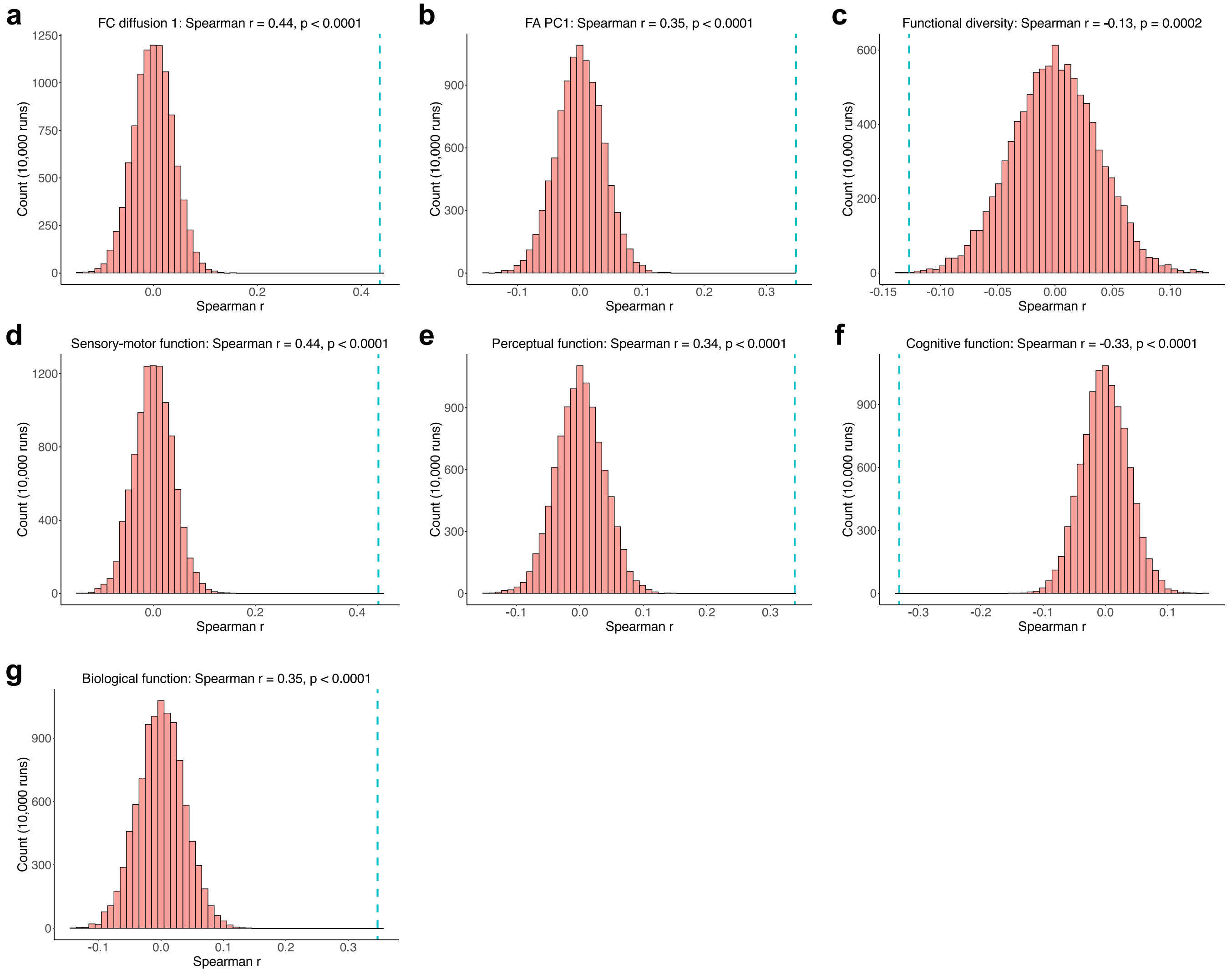

Fig. S9: Null model tests for seven significant macroscale functional gradients that correlate with structure-function correspondence. All null model tests were conducted by randomly assigning computed functional metrics to parcels over 10,000 runs. The x-axis indicates the Spearman r between the select functional metric from Fig. 5 and SF R^2 by parcel. Null model tests for **a** first component of the FC-Neurosynth diffusion map, **b** first FA-Neurosynth principal component, **c** functional diversity, **d** sensory-motor function, **e** cognitive function, **f** perceptual function, and **g** biological function. All are shown to be significant. Source data are provided as a Source Data file.
