# Supplementary material for: Mapping the structure-function relationship along macroscale gradients in the human brain
Source: Nat Commun. 2024 Aug 16;15:7063. doi: 10.1038/s41467-024-51395-6 (PMC11329792; doi:10.1038/s41467-024-51395-6)
Supplement: Supplementary file 3 — Reporting Summary [file 41467_2024_51395_MOESM3_ESM.pdf]

nature portfolio

Corresponding author(s): Evan Collins

Last updated by author(s): Jul 9, 2024

Reporting Summary

Nature Portfolio wishes to improve the reproducibility of the work that we publish. This form provides structure for consistency and transparency in reporting. For further information on Nature Portfolio policies, see our Editorial Policies and the Editorial Policy Checklist.

Statistics

For all statistical analyses, confirm that the following items are present in the figure legend, table legend, main text, or Methods section.

n/a	Confirmed	
☐	☒	The exact sample size (*n*) for each experimental group/condition, given as a discrete number and unit of measurement
☐	☒	A statement on whether measurements were taken from distinct samples or whether the same sample was measured repeatedly
☐	☒	The statistical test(s) used AND whether they are one- or two-sided<br>*Only common tests should be described solely by name; describe more complex techniques in the Methods section.*
☐	☒	A description of all covariates tested
☐	☒	A description of any assumptions or corrections, such as tests of normality and adjustment for multiple comparisons
☐	☒	A full description of the statistical parameters including central tendency (e.g. means) or other basic estimates (e.g. regression coefficient) AND variation (e.g. standard deviation) or associated estimates of uncertainty (e.g. confidence intervals)
☐	☒	For null hypothesis testing, the test statistic (e.g. *F*, *t*, *r*) with confidence intervals, effect sizes, degrees of freedom and *P* value noted<br>*Give P values as exact values whenever suitable.*
☒	☐	For Bayesian analysis, information on the choice of priors and Markov chain Monte Carlo settings
☐	☒	For hierarchical and complex designs, identification of the appropriate level for tests and full reporting of outcomes
☐	☒	Estimates of effect sizes (e.g. Cohen's *d*, Pearson's *r*), indicating how they were calculated

Our web collection on statistics for biologists contains articles on many of the points above.

Software and code

Policy information about availability of computer code

Data collection

The raw dMRI data and processed dMRI data in MNI space can be accessed at https://db.humanconnectome.org/ and https://brain.labsolver.org/hcp_ya.html, respectively, under restricted access to preserve subject privacy. Access to HCP can obtained by account creation and signing a data use agreement described at https://www.humanconnectome.org/study/hcp-young-adult/data-use-terms. The raw Neurosynth data can be accessed at https://github.com/neurosynth/neurosynth-data. LIWC2015 data is not publicly available due to licensing reasons; users can ascertain access details at https://www.liwc.app/. Except for LIWC2015, the Yale Brain Atlas data and all raw and processed data to replicate methods and results of this paper are available at https://github.com/evancollins1/brain_structure_function. Yale Brain Atlas can be interactively viewed at https://yalebrainatlas.github.io/YaleBrainAtlas/. Source data used to generate figures are provided in the Source Data file.

As detailed in our GitHub repository, the following R (v4.3.1) packages (and their associated version numbers) were used for this project:
dplyr=1.1.2
plotly=4.10.2
ggplot2=3.4.3
Rmisc=1.5.1
png=0.1.8
grid=4.3.1
ggpubr=0.6.0
reactable=0.4.4
reactablefmtr=2.0.0
readobj=0.4.1

svMisc=1.2.3
httr=1.4.6
easyPubMed=2.13
word2vec=0.3.4
reticulate=1.30
mclust=6.0.0
tidyverse=2.0.0
ggseg3d=1.6.3
lsa=0.73.3
corrplot=0.92
ggbreak=0.1.2
patchwork=1.1.2
Rtsne=0.16
ggrepel=0.9.3
ggcharts=0.2.1
igraph=1.5.0
quanteda=3.3.1
MASS=7.3.60
factoextra=1.0.7
ggtext=0.1.2
rmatio=0.19.0
leaps=3.1
car=3.1.2
igraph=1.5.0
openxlsx=4.2.5.2

The following Python (3.10.12) packages (and their associated version numbers) were used for this project:
pandas=1.5.3
numpy=1.23.5
scipy=1.11.3
pip=22.3.1
matplotlib=3.8.2
pytorch=2.2.0
nilearn=0.10.2
pip:
plotly==5.15.0
kaleido==0.2.1
neuroquery==1.0.4
nibabel==5.2.0
scikit-learn==1.3.2

Data analysis

All data processing and analysis code is available on GitHub:
https://github.com/evancollins1/brain_structure_function.
Justification of data analysis approaches can be found in the Methods section of manuscript.

For manuscripts utilizing custom algorithms or software that are central to the research but not yet described in published literature, software must be made available to editors and reviewers. We strongly encourage code deposition in a community repository (e.g. GitHub). See the Nature Portfolio guidelines for submitting code & software for further information.

Data

Policy information about availability of data

All manuscripts must include a data availability statement. This statement should provide the following information, where applicable:

- Accession codes, unique identifiers, or web links for publicly available datasets
- A description of any restrictions on data availability
- For clinical datasets or third party data, please ensure that the statement adheres to our policy

The raw dMRI data and processed dMRI data in MNI space can be accessed at https://db.humanconnectome.org/ and https://brain.labsolver.org/hcp_ya.html, respectively, under restricted access to preserve subject privacy. Access to HCP can obtained by account creation and signing a data use agreement described at https://www.humanconnectome.org/study/hcp-young-adult/data-use-terms. The raw Neurosynth data can be accessed at https://github.com/neurosynth/neurosynth-data. LIWC2015 data is not publicly available due to licensing reasons; users can ascertain access details at https://www.liwc.app/. Except for LIWC2015, the Yale Brain Atlas data and all raw and processed data to replicate methods and results of this paper are available at https://github.com/evancollins1/brain_structure_function. Yale Brain Atlas can be interactively viewed at https://yalebrainatlas.github.io/YaleBrainAtlas/. Source data used to generate figures are provided in the Source Data file.

Research involving human participants, their data, or biological material

Policy information about studies with human participants or human data. See also policy information about sex, gender (identity/presentation), and sexual orientation and race, ethnicity and racism.

Reporting on sex and gender	Sex information collected for rsfMRI study. Sex was based on that assigned at birth. Reported in manuscript.<br>Sex information sourced from HCP for tractography and cortical thickness data. Reported in manuscript.
Reporting on race, ethnicity, or other socially relevant groupings	N/A
Population characteristics	Mean age and range reported for rsfMRI, tractography (from HCP), and cortical thickness (from HCP) data.
Recruitment	For the rfMRI study, the healthy controls (i.e., all participants included for this paper) were recruited via a flyer (that was approved by the IRB) that was posted at various locations; they then contacted us. Each participant was compensated $40. Informed consent was obtained from each participant.
Ethics oversight	IRB (HIC#s) associated with this study are 1003006485 & 0702002395

Note that full information on the approval of the study protocol must also be provided in the manuscript.

Field-specific reporting

Please select the one below that is the best fit for your research. If you are not sure, read the appropriate sections before making your selection.

☒ Life sciences ☐ Behavioural & social sciences ☐ Ecological, evolutionary & environmental sciences

For a reference copy of the document with all sections, see nature.com/documents/nr-reporting-summary-flat.pdf

Life sciences study design

All studies must disclose on these points even when the disclosure is negative.

Sample size	Besides rsfMRI study, all sample sizes are 100+, which have a theoretical statistical power of >94% (Halsey et al. Nature Methods (2015)). For the rsfMRI cohort, we chose a sample size of 34, comparable to similar prior studies and sufficiently large for statistical tests when stricter critical thresholds were set.
Data exclusions	Only data exclusion comes during our preparation of Figure 5. We detail this in Methods: "We only considered the top ten subset of activated functional terms per parcel to reduce the effect of small type I errors from imperfect topic analysis in Neurosynth. The mean number of activated functional terms per parcel was ~55, so taking the top 10 terms reflects the top ~20% of functional terms. We found this amount optimized correlative performance, suggesting some ideal representation of function in each parcel. Note, however, that correlative significance was still observed for relationships when all activated terms were considered."
Replication	All analyses to replicate results included in GitHub repository. Null model tests run for key correlations (Figure S9).
Randomization	Allocation to experimental and control groups was not needed for this study.
Blinding	Not relevant to our study, as all subjects underwent same rsfMRI protocol.

Reporting for specific materials, systems and methods

We require information from authors about some types of materials, experimental systems and methods used in many studies. Here, indicate whether each material, system or method listed is relevant to your study. If you are not sure if a list item applies to your research, read the appropriate section before selecting a response.

Materials & experimental systems

n/a	Involved in the study	
☒	☐	Antibodies
☒	☐	Eukaryotic cell lines
☒	☐	Palaeontology and archaeology
☒	☐	Animals and other organisms
☒	☐	Clinical data
☒	☐	Dual use research of concern
☒	☐	Plants

Methods

n/a	Involved in the study	
☒	☐	ChIP-seq
☒	☐	Flow cytometry
☐	☒	MRI-based neuroimaging

Plants

Seed stocks: N/A

Novel plant genotypes: N/A

Authentication: N/A

Magnetic resonance imaging

Experimental design

Design type: Resting-state (data was sourced from Neurosynth and NeuroQuery data repositories)

Design specifications: Two to three 5-minute resting-state runs were acquired for each of the 34 subjects. The data was ultimately transformed into Yale Brain Atlas space. Mean rsfMRI activations across the entire time course were computed for each parcel. Finally, the connectivity matrix was obtained using the Fisher transformation of the Pearson correlation coefficient for each pair of parcels. The final output from this processing was a rsfMRI pairwise correlation connectivity matrix of size 696 by 696 (parcels) for each of the 34 healthy subjects.

Behavioral performance measures: N/A

Acquisition

Imaging type(s): Functional

Field strength: 3-Tesla

Sequence & imaging parameters: For rsfMRI data acquisition, imaging was performed on a 3-Tesla Siemens Trio scanner (Siemens Medical Systems, Erlangen, Germany) using a 64-channel head coil for 34 subjects (17 females, 17 males; mean age 33 years; age range 18 to 55 years) (IRB HIC#s 1003006485 & 0702002395). Each subject was positioned in the coil, and head movements were minimized with pillows. After a three-plane localizer, a high-resolution whole-brain T1-weighted three-dimensional magnetization-prepared rapid gradient echo volume scan was acquired for multisubject registration. An oblique axial T1 image parallel to the anterior commissure–posterior commissure (AC-PC) line was acquired. Two to three 5-minute resting-state runs were then acquired.

Area of acquisition: Whole-brain

Diffusion MRI: [x] Used [ ] Not used

Parameters: Sourced from HCP. Structural data was acquired from Yeh (2022), which includes processed tractograms derived from dMRI data for 1,065 young adult subjects (575 females, 490 males; mean age 28.74 years; age range from 22 to 37 years). The dMRI data originated from HCP and was converted to the DSI Studio file format. Yeh reconstructed the dMRI data in the MNI common space using q-space diffeomorphic reconstruction. For our analysis, a deterministic fiber tracking algorithm was used, and a seeding region was placed across the whole brain in DSI Studio. The anisotropy threshold was 0.05208 (0.6 × Otsu's threshold). The angular threshold was 60 degrees. The step size was 0.5 voxels. Tracks with length shorter than 10 mm or longer than 300 mm were discarded. A total of 1,000,000 streamlines were generated for each subject, and two iterations of topology-informed pruning were performed to remove false positive streamlines. The SC matrix for WM streamline count was calculated for each subject by counting streamlines with endpoints in distinct parcel pairs.

Preprocessing

Preprocessing software: Yale BioImage Suite

Normalization: All scans were converted from Digital Imaging and Communication in Medicine format to NIfTI format. To take the individual subject data into a common reference space, we calculated sequential registrations within Yale BioImage Suite: a linear registration between the individual subject's rsfMRI image and their anatomical image, and a nonlinear registration between the subject's anatomical image and the standard whole-brain template (MNI152 1 mm template).

Normalization template: MNI152

Noise and artifact removal: All scans were converted from Digital Imaging and Communication in Medicine format to NIfTI format. During the conversion process, six images were omitted to allow the signal to attain steady-state equilibrium between radio frequency pulsing and relaxation. Resting-state images were motion corrected using SPM8. Trials with linear motion that had a displacement more

	than 2 mm or rotation more than 3° were omitted. Artefact time courses related to cardiovascular and breathing effects (i.e., mean time courses from CSF and white matter) and global signal were regressed out. Bandpass filtering with a passband between 0.008 and 0.12 Hz was applied such that the output time course accurately represented the hemodynamic change of the BOLD signal.
Volume censoring	Resting-state images were motion corrected using SPM8. Trials with linear motion that had a displacement more than 2 mm or rotation more than 3° were omitted.

Statistical modeling & inference

Model type and settings	Mean rsfMRI activations across the entire time course were computed for each parcel. Finally, the connectivity matrix was obtained using the Fisher transformation of the Pearson correlation coefficient for each pair of parcels. Hence, the final output from this processing was a rsfMRI pairwise correlation connectivity matrix of size 696 by 696 (parcels) for each of the 34 healthy subjects.
Effect(s) tested	Resting-state connectivity patterns

Specify type of analysis: ☐ Whole brain ☒ ROI-based ☐ Both

Anatomical location(s): Yale Brain Atlas (YBA) parcellation

Statistic type for inference (See Eklund et al. 2016)	Cluster-wise
Correction	Bonferroni, where applicable to mitigate any selection biases

Models & analysis

n/a	Involved in the study
☐	☒ Functional and/or effective connectivity
☐	☒ Graph analysis
☐	☒ Multivariate modeling or predictive analysis

Functional and/or effective connectivity	See Methods subsections entitled "Structural connectivity", "Neurosynth-derived functional connectivity", "NeuroQuery-derived functional connectivity", and "rsfMRI-derived functional connectivity".
Graph analysis	See Methods subsection entitled "Diffusion map and spectral clustering".
Multivariate modeling and predictive analysis	See Methods subsection entitled "Linear regression model selection".
